# Examining Community Engagement Strategies Used in the Climate Impact on Lung Cancer via Exposure to Radon (CLOVER) Study

**DOI:** 10.3390/ijerph23081072

**Published:** 2026-08-18

**Authors:** Mary Srivastava, Andrea Thoumi, Yadurshini Raveendran, Phillip Gibson, Jules Iradukunda, Ashwini Joshi, Suur D. Ayangeakaa, Jeffrey M. Clarke, Amie Koch, Junfeng (Jim) Zhang, Tomi Akinyemiju

**Affiliations:** 1 Department of Population Health Sciences, Duke University School of Medicine, Durham, NC 27701, USA; mary.srivastava@duke.edu (M.S.);; 2 St. Jude Children’s Research Hospital, Memphis, TN 38105, USA; 3 North Carolina Department of Health and Human Services, Raleigh, NC 27612, USA; 4 Duke Global Health Institute, Durham, NC 27708, USA; 5 Duke Cancer Institute, Duke University School of Medicine, Durham, NC 27710, USA; 6 Duke School of Nursing, Duke University, Box 3322, Durham, NC 27710, USA; 7 Nicholas School of the Environment, Duke University, Durham, NC 27708, USA

**Keywords:** radon, community-engaged research, address-based sampling, health disparities

## Abstract

**Highlights:**

**Public health relevance—How does this work relate to a public health issue?**

- Radon is the second leading cause of lung cancer in the United States, and disparities in radon awareness and testing contribute to environmental health inequities in North Carolina.
- Underrepresentation of diverse populations in environmental health research limits understanding of inequities in radon exposure and the development of equitable prevention strategies.

**Public health significance—Why is this work of significance to public health?**

- Evidence comparing recruitment strategies for enrolling racially and ethnically diverse populations into environmental health studies remains limited.
- This study demonstrates that community-engaged recruitment can improve participation among racially and ethnically diverse populations and increase completion of radon testing.

**Public health implications—What are the key implications or messages for practitioners, policy makers, and/or researchers in public health?**

- Trusted community partnerships and culturally responsive outreach can strengthen recruitment and participation in environmental health research.
- Community-engaged approaches should be incorporated into radon education, testing, and research initiatives to promote equitable participation and reduce lung cancer disparities.

**Abstract:**

Background: Residential radon exposure is a leading risk factor for lung cancer, and climate change may exacerbate this risk by increasing radon entry into homes. In North Carolina (NC), disparities in lung cancer outcomes and low radon awareness disproportionately affect racially and ethnically diverse and low-income populations. However, little is known about how use of conventional address-based sampling (ABS) compares with community-engaged recruitment strategies for enrolling historically underrepresented populations into such research. Community-engaged approaches are used to improve participation in environmental health research, yet few studies compare their effectiveness with traditional approaches in NC. This manuscript compares recruitment outcomes between ABS and community-engaged approaches within the CLOVER study to address this gap. Materials and Methods: The Climate Impact on Lung Cancer via Exposure to Radon (CLOVER) study is a cross-sectional study examining climate-impacted radon exposure and lung cancer risk in NC. Participants were recruited using either ABS through a commercial address database or targeted community-engaged approaches implemented through partnerships with community organizations and culturally responsive, in-person outreach events. After providing informed consent, participants completed a household survey and a 7-day home radon test. Recruitment outcomes—consent, survey completion, and radon test return—were stratified by race and ethnicity and compared across strategies. Results: Of 236 consented participants, community-engaged recruitment enrolled a higher proportion of Non-Hispanic (NH) Black, Hispanic/Latino, and American Indian participants than ABS (57.3% vs. 41.9%). Community-engaged recruitment also had a higher consent rate than ABS (10.2% vs. 1.3%). Of the 145 participants who completed the survey, ABS participants completed surveys at a higher rate than community-engaged participants (73.3% vs. 54.7%), while of the 86 who returned the radon test kits, community-engaged participants did so at twice the rate of ABS participants (44.7% vs. 22.1%). Conclusions: Community-engaged recruitment enrolled a more racially and ethnically diverse sample and achieved higher consent and return rates than ABS, though ABS participants completed surveys at a higher rate. These findings highlight the importance of community partnerships and in-person recruitment strategies to improve participation of historically underrepresented populations in environmental health research and support equitable approaches to radon mitigation and lung cancer prevention.

## 1. Introduction

Radon, a radioactive gas derived from the decay of elements such as uranium, thorium, and radium, is the second leading cause of lung cancer in the United States (U.S.) and is responsible for an estimated 21,000 deaths annually [1,2]. Climate change may compound this epidemiological risk by altering precipitation patterns, hydrological processes, and soil conditions that can influence radon entry into homes, particularly in regions already prone to elevated indoor radon levels [2,3,4]. In North Carolina (NC), lung cancer incidence exceeds the national average (62.1 per 100,000 vs. 52.8 per 100,000), and long-standing racial and ethnic disparities in radon exposure, screening, and mitigation compound this cancer burden [5,6,7].

Compared to non-Hispanic (NH) White patients, many historically marginalized patients with lung cancer experience later diagnoses and disparities in receipt of guideline-concordant care. For example, non-Hispanic (NH) Black patients are less likely to be diagnosed at an early stage (25.4% of Black patients vs. 28.8% of White patients), receive surgical treatment (16.4% of NH Black patients vs. 20.0% of NH White patients), and have lower five-year survival rates (28.1% of NH Black patients vs. 29.0% of NH White patients) [7]. Similarly, American Indian and Hispanic/Latino patients are less likely to be diagnosed early (22.4% of American Indian patients and 23.4% of Hispanic/Latino patients vs. 28.8% of NH White patients), and American Indian patients are less likely to receive surgical treatment for lung cancer in NC (15.6% of American Indian patients vs. 20.0% of NH White patients) [7]. These disparities reflect broader structural and social inequities that influence access to cancer prevention, screening, and treatment [8,9]. Similar disparities by race, ethnicity, and income exist in awareness of radon as a lung cancer risk factor. For example, in a 2019 survey of NC residents, 86% of Hispanic residents, 63% of NH Black residents (vs. 35% of NH White residents), and 88% of residents below the poverty level (vs. 29% of residents above the poverty level) had no awareness of radon gas as an environmental risk factor [10]. Despite marginalized communities exhibiting the highest need for radon testing, testing and mitigation patterns differ across socioeconomic and demographic groups, with testing rates highest among affluent and college-educated households and lowest in census tracts with greater socioeconomic disadvantage and larger NH Black populations [11]. Together, these findings highlight disparities in lung cancer outcomes and radon awareness, underscoring the need for equitable approaches to prevention, outreach, and mitigation.

In response to this gap, the Climate Impact on Lung Cancer via Exposure to Radon (CLOVER) study was established in 2023 to examine how changing climate patterns influence residential radon exposure, lung cancer risk and outcomes, and the potential impact of radon mitigation across racial/ethnic and sociodemographic populations of NC. CLOVER draws on secondary radon testing and sociodemographic data alongside primary data collection designed to supplement existing data in populations with historically low radon testing rates; this effort aims to test radon levels in 1000 households stratified by race/ethnicity, geographic region, and homeownership across all 100 counties. Because representative enrollment is essential to this goal, CLOVER employed multiple recruitment strategies, including address-based sampling (ABS) and community-engaged approaches, to reach historically underrepresented populations.

This dual recruitment approach reflects a broader, unresolved challenge in environmental health research: populations disproportionately affected by environmental exposures that result in elevated radon exposure in the home have been historically underrepresented in environmental health research, limiting the ability of environmental health studies to generate representative exposure data, accurately characterize exposure-related disease risk, and produce findings that are generalizable across diverse populations [12]. Community-engaged research practices, rooted in equity and social justice frameworks, are effective strategies for building trust and increasing participation among historically underrepresented populations [13,14,15]. ABS, by contrast, is a widely used probability-based sampling method that offers systematic population-level coverage and is considered a methodological standard for household surveys in the U.S. [16]. However, no studies have compared the effectiveness of community-engaged recruitment strategies to ABS for recruiting historically underrepresented populations into environmental health studies in NC. Recruitment strategy for CLOVER will shape the representativeness of radon exposure data used to model the effects of climate change on radon levels and lung cancer risk across diverse populations, with implications for recruitment practice in environmental health research more broadly. This paper describes and compares recruitment outcomes associated with ABS and community-engaged recruitment approaches used to enroll NH Black, Hispanic/Latino, and American Indian participants into the CLOVER study and examines potential factors contributing to differences in participation outcomes.

## 2. Materials and Methods

### 2.1. Study Design

The CLOVER study is a cross-sectional study conducted in NC to characterize climate-related residential radon exposure and its association with lung cancer risk. Participants were recruited using a stratified sampling approach to complete a household survey and a 7-day continuous radon test. The study protocol was approved by Duke University Institutional Review Board (IRB Pro 00113240).

### 2.2. Community Partnerships and Engagement Strategy

Community-engaged recruitment was supported by partnerships with community and state-level stakeholders and was integral to the design and implementation of recruitment strategies. Key partners included the Duke Cancer Institute Community Outreach and Engagement (COE) Community Health Ambassadors (CHAs) and Community Advisory Council (CAC), whose members represent diverse geographic, racial, and ethnic communities across NC; the North Carolina Department of Health and Human Services (NCDHHS), including the state Radon Program coordinator; and community-based organizations, faith-based organizations, and tribal communities (Supplementary Table S2). Some partnerships were previously established (e.g., CHAs, tribal councils) and others were newly established for this study.

These partners contributed to study design by informing development of the study protocol, reviewing and refining culturally appropriate recruitment materials, and advising on dissemination strategies for radon test kits and participant-facing study information. Partnerships with NCDHHS also supported alignment of the study activities with existing public health infrastructure and radon education initiatives. Engagement with partners varied by role, with regular monthly meetings held with NCDHHS, CHAs, and CAC members, while collaborations with community partner organizations occurred on an ad hoc basis.

In recruitment, partners played an active role in identifying appropriate venues for outreach, facilitating introductions to local health departments, community organizations, and community leaders, and co-hosting recruitment events. Partners also advised on culturally appropriate messaging, assisted with event coordination, and, in some cases, supported on-site engagement by encouraging participation and answering questions. Linkage to radon mitigation resources for households with elevated radon levels was supported by NCDHHS. Together, these roles enhanced the cultural relevance, credibility, and reach of community-engaged recruitment efforts.

### 2.3. Study Population

The study population consisted of adults (≥18 years) residing in NC households who were eligible to provide informed consent, complete the survey in English or Spanish, and complete the radon test kit. Exclusion criteria included being younger than 18, living outside of NC, or being unable to complete the survey due to language or cognitive barriers.

### 2.4. Participant Recruitment

Participants were recruited using two approaches: ABS and community-engaged recruitment.

Address-Based Sampling (ABS) Recruitment: From November 2023 to July 2024, participants were recruited via ABS using a commercial database provided by Marketing Systems Group (MSG). ABS sampling was stratified by geographic region (mountains, piedmont, and coastal plains), homeownership status, race (NH Black vs. non-Black), and rural/urban classification to increase representation among priority populations, including NH Black residents and residents in the mountain region. NH Black residents were oversampled across all geographic regions, and non-Black residents in the mountain region were also oversampled. In this study, ABS was defined as residential and email address sampling. Eligible NC households with available email addresses (~53%) received an initial study invitation with up to four multi-modal reminder messages. Non-respondents or households without email addresses were contacted by phone. Households not reached through email or telephone were mailed a recruitment postcard containing study details followed by a reminder postcard three weeks later. Individuals who agreed to participate completed the study survey either by telephone or online via REDCap and were subsequently mailed a radon test kit for completion.

**Figure 1:**
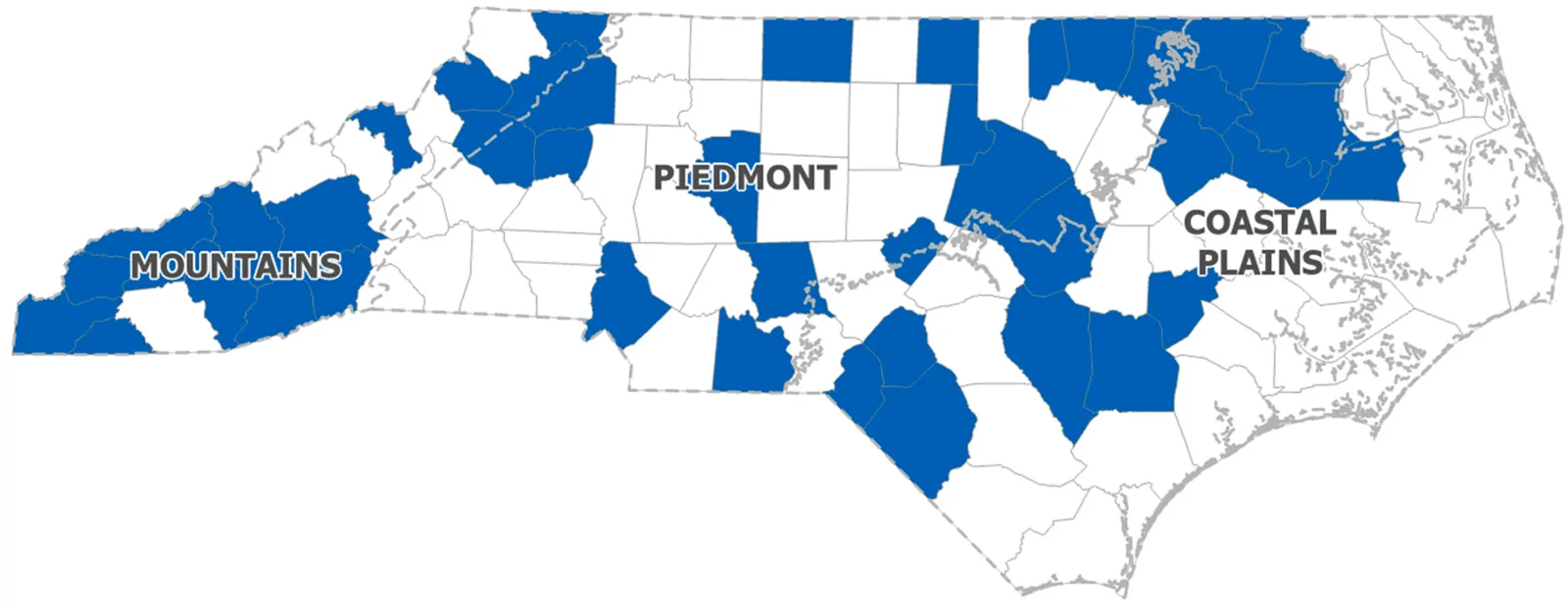
Geographic distribution of target counties selected for community-engaged recruitment in North Carolina. Counties were selected based on demographic and radon exposure criteria and are shown across the mountains, piedmont, and coastal plains regions of the state.

Community-Engaged Recruitment: From August 2024 to May 2025, participants were recruited using community-engaged approaches in partnership with community-based organizations, faith-based institutions, tribal communities, CHAs, and the CAC. Recruitment efforts prioritized NH Black, Hispanic/Latino, and American Indian residents, as well as individuals living in high-radon counties. Forty target counties were selected from all 100 NC counties based on EPA radon zone designation, elevated radon concentrations, and demographic criteria reflecting populations disproportionately affected by lung cancer disparities. Detailed selection criteria are provided in Supplementary Table S1. These 40 counties were classified by geographic region (mountains, piedmont, and coastal plains) to support regionally tailored recruitment strategies (Figure 1). Average demographic composition, radon exposure levels, and EPA zone designations for each region are presented in Table 1. Recruitment strategies included participant enrollment and distribution of radon test kits at community events (e.g., health fairs, cultural festivals, and church gatherings). Additionally, informational sessions tailored to cultural and linguistic needs were conducted, including Spanish-language sessions, and trusted community advocates (e.g., CHAs, CAC members, faith leaders, and tribal leaders) were engaged to share study information. Recruitment in the mountain region was paused November 2024–January 2025 due to Hurricane Helene.

### 2.5. Data Collection

Participants completed a household survey and 7-day continuous, short-term radon test kit provided by Air Check LLC (Mills River, NC, USA). Surveys were administered via telephone or online via REDCap. Radon tests were distributed in person at community events or mailed directly to participants’ addresses and processed by Air Chek LLC (Mills River, NC, USA). Test results were shared with participants and the study team. Participants with radon levels ≥ 4 pCi/L were provided a second test for confirmation, and those with confirmed elevated radon levels (defined as an average radon concentration of ≥4 pCi/L across both tests) were referred to NCDHHS for mitigation support. All participants who returned a completed radon test received a thank-you postcard. Participants received a USD 10 VISA gift card for completing the survey and an additional USD 10 VISA gift card for returning a completed radon test.

### 2.6. Data Analysis

Analyses compared recruitment outcomes across ABS and community-engaged approaches, including recruitment yield, demographic diversity, and geographic reach.

Frequencies and proportions were calculated to describe the number and percentage of participants who (1) consented to the study, (2) completed the survey, and (3) returned radon test results, stratified by recruitment approach. Recruitment success rates were calculated as the number of participants who consented divided by the total number of contact attempts (calls, mailings, and emails for ABS recruitment methods; community event attendees for community-engaged recruitment). Survey completion and radon test kit return rates were calculated among participants who consented to the study within each recruitment strategy. Cross-tabulations were used to examine differences in demographic composition (e.g., race/ethnicity) between recruitment approaches, with particular attention to representation of NH Black, Hispanic/Latino, and American Indian participants. Regional recruitment data were summarized using frequencies to identify geographic variation in participation across the three community-engaged recruitment regions (mountains, piedmont, and coastal plains).

Survey data were exported from REDCap into R (version 4.4.2) for analysis. Descriptive statistics were used to summarize participant characteristics, including age, sex, race/ethnicity, educational attainment, employment status, and health insurance coverage. Missing or incomplete survey data were examined prior to analysis; participants with missing demographic information were excluded from specific analyses but retained in the overall dataset.

## 3. Results

Community-engaged recruitment was conducted in partnership with 18 unique community organizations across NC. Of these, 12 were located in counties selected as target counties for community-engaged recruitment (Supplemental Table S2). The primary method of community-engaged recruitment was participation in community events hosted by partner organizations or identified through CHAs and CAC members. The study team participated in 25 community events between August 2024 and May 2025, including health fairs, cultural festivals, and church-based outreach activities, with an average of 56 radon test kits distributed per event. Events were most frequently conducted in the piedmont (n = 14), followed by the coastal plains (n = 10) and the mountains (n = 1).

In total, 236 participants consented, 145 participants completed the survey, and 86 radon test results were obtained (Table 2). ABS recruitment methods resulted in 86 participants consenting out of 6648 contact attempts, corresponding to a 1.3% success rate. Community-engaged recruitment resulted in 150 participants consenting from approximately 1420 distributed radon test kits, with a success rate of 10.2%. Survey completion rates were high among participants recruited via ABS (73.3%) compared to community-engaged approaches (54.7%) (Table 2). In contrast, radon test return rates were higher among participants recruited through community-engaged approaches (44.7%) compared to ABS (22.1%) (Table 2). These findings demonstrate differing patterns of engagement across recruitment approaches, with ABS yielding higher survey completion rates and community-engaged approaches producing higher radon test return rates. Differences in demographic distribution of participants were observed across recruitment approaches. Higher proportions of NH Black, Hispanic/Latino, and American Indian participants were recruited via community-engaged approaches compared to ABS (Table 2). Community-engaged recruitment was particularly effective in reaching NH Black participants, with 44.0% of NH Black participants recruited through community-engaged recruitment compared 37.2% via ABS. Similarly, community-engaged recruitment was more effective for reaching American Indian participants, with 8.0% of American Indian participants recruited through community-engaged approaches compared to 1.2% via ABS (Table 2).

Sociodemographic characteristics of participants who completed the survey (n = 145) are summarized in Table 3. Participants had a mean age of 49.8 years (SD = 15.8), with most participants identifying as female (64.8%). The sample included a high proportion of NH Black (37.2%), Asian (23.4%), and American Indian (8.3%) populations. Most participants held a university degree or higher (59.3%), with an additional 24.8% reporting a college, technical, or associate degree. Employment status and health insurance varied, with most participants being employed part-time or full-time (64.1%), and most participants reported private insurance (57.2%) or public insurance (32.4%).

## 4. Discussion

In a cross-sectional study examining residential radon exposure influenced by climate change and lung cancer outcomes in NC, we compare the effectiveness of address-based sampling (ABS) recruitment and community-engaged recruitment strategies in enrolling populations disproportionately affected by radon exposure. Community-engaged recruitment demonstrated substantially higher success rates than ABS recruitment for the percentage of participants who consented and the percentage of radon test kits returned, while ABS recruitment yielded higher survey completion rates. Community-engaged approaches were more effective for enrolling racially and ethnically diverse participants, particularly NH Black and American Indian populations, compared with ABS recruitment. The lower percentage of surveys completed among community-engaged participants may reflect logistical factors inherent to event-based recruitment, where participants often enroll in person but complete the survey later, increasing opportunities for disengagement or loss to follow-up.

Several social and structural mechanisms likely explain the higher performance of community-engaged recruitment compared to ABS recruitment. Trust, established through partnerships with faith-based organizations, tribal leadership, CHAs, and the CAC, may have contributed to increased willingness to participate, consistent with evidence that established community relationships and community-led recruitment can facilitate participation in environmental health research [15,18]. While some strategies, including on-site assistance with study participation and distribution of study materials, were applied broadly across recruitment settings, tailored approaches were used to engage racial and ethnic minority populations through culturally relevant and community-specific methods. Our tailored approach in the recruitment of American Indian communities prioritized collaboration with tribal leadership [19,20], including engagement at community-centered events such as Powwows and Homecomings, which are longstanding gathering points within tribal communities. A high proportion of our outreach to NH Black communities was conducted through churches, with consent and survey completion facilitated in partnership with church leadership following services, consistent with prior research highlighting the role of faith-based institutions in facilitating participation among NH Black communities in health research [21]. Engagement with Hispanic/Latino communities relied on partnerships with CBOs such as El Centro Hispano, participation in Spanish-language community events, and implementation of bilingual staffing models and materials [22]. Community-engaged recruitment reduced logistical barriers by providing radon test kits in-person, offering live assistance during events, and minimizing delays associated with mail delivery, factors that likely contributed to higher test return rates than ABS recruitment, consistent with prior work showing that in-person distribution through trusted local institutions can improve radon test kit return rates relative to more passive distribution approaches [23].

Beyond improving recruitment outcomes, these findings have important implications for environmental health research. Studies examining environmental health exposures depend on representative participation to accurately characterize exposure distributions, identify disparities across populations, and generate findings that are applicable to communities most affected by environmental risks [9,12,24]. Historically, populations experiencing the greatest burden of environmental exposures have often been underrepresented in environmental health research, limiting the ability of studies to fully characterize exposure patterns and associated health disparities [12]. Because the broader CLOVER study seeks to estimate the effects of climate change on residential radon exposure, lung cancer risk, and the potential benefits of radon mitigation across populations defined by race/ethnicity and social determinants of health, representative recruitment is fundamental to the validity of these analyses. By improving participation among historically underrepresented populations, community-engaged recruitment may strengthen environmental exposure assessment and enhance the evidence used to inform equitable environmental health policies and prevention strategies [12,25].

Findings from this study align with a growing body of literature demonstrating that community-engaged approaches can enhance recruitment and participation among underserved and minority populations in environmental health research [15,18,25]. Prior studies show that engagement strategies incorporating partnerships with community organizations, place-based outreach, and culturally responsive communication are more effective at reaching populations disproportionately affected by environmental exposures, including air pollution, lead, and housing-related hazards [26,27,28]. Evidence more broadly from health and medical research suggests that recruitment efforts grounded in community context improve participation by addressing barriers related to trust, risk awareness, and accessibility, limitations commonly observed with mail-based or clinic-centered recruitment alone [29]. This pattern extends to environmental exposure and cumulative risk research specifically, where community engagement has similarly been shown to improve study reach and relevance [25]. Collectively, this literature supports the patterns observed in the CLOVER study, reinforcing the role of community engagement as a critical methodological approach for improving study sample representativeness in environmental health research. Our findings extend this literature by demonstrating that community-engaged recruitment can more effectively engage historically underrepresented populations in a statewide study examining the effect of climate change on radon exposure and lung cancer outcomes, highlighting the importance of incorporating community-engaged approaches when collecting data in environmental health research.

This study has several limitations. Recruitment in the mountain region was disrupted by Hurricane Helene, limiting event-based outreach and illustrating the broader challenge of conducting community-engaged research in geographically dispersed regions, where infrastructure or transportation may impede participation. Recruitment strategies were not implemented concurrently; ABS and community-engaged approaches occurred in different time periods, raising the possibility that seasonal differences may have influenced opportunities for community engagement, participant responsiveness, and interest in radon testing. Future studies comparing recruitment approaches would benefit from concurrent implementation or designs that allow seasonal effects to be modeled and separated from recruitment strategy effects. Survey respondents overall had high educational attainment, potentially limiting generalizability to more socioeconomically disadvantaged populations; a demographic skew toward older, higher-income participants has similarly been observed in other radon testing initiatives using clinic-based distribution models [23]. Findings may not generalize beyond NC, given that the state’s radon geology, demographics, and community infrastructure (e.g., tribal governance structures, faith-based networks, and CHA programs) shaped the community-engaged strategies used and the small subgroup sample sizes (e.g., Hispanic/Latino, American Indian) limit the precision of subgroup-specific comparisons and the feasibility of formal statistical testing. Finally, recruitment success rates are not directly comparable across strategies, as ABS success was calculated relative to individual contact attempts, while community-engaged success was calculated relative to event attendees, and both were measured as raw counts rather than by more granular process measures (e.g., partnership maturity, event size, or staff capacity). Future research should explore measures that would allow more direct comparison of recruitment effectiveness across fundamentally different recruitment modalities.

Overall, these findings demonstrate that community-engaged recruitment methods are more effective than ABS recruitment approaches for enrolling diverse populations into environmental health studies. These differences are likely driven by factors such as trust-building, culturally tailored engagement, and reduced logistical barriers to participation. Improving participation among populations historically underrepresented in environmental health research has implications beyond recruitment alone and can strengthen the representativeness of environmental exposure data and improve understanding of the disparities in radon exposure and lung cancer risk. As climate change increasingly shapes radon infiltration patterns, these results underscore the need for forward-looking policies that strengthen community partnerships and expand access to radon testing and mitigation in NC.

## 5. Conclusions

This paper provides novel empirical evidence comparing community-engaged and ABS recruitment approaches for enrolling racially and ethnically diverse populations in an observational radon exposure study in NC, adding to a limited evidence base on how recruitment strategy shapes representation and generalizability in radon exposure research. By leveraging trusted community partnerships, culturally responsive communication, and in-person engagement, community-engaged approaches were effective in reaching NH Black, Hispanic/Latino, and American Indian populations, groups historically underrepresented in environmental health research. These findings have practical implications for researchers designing recruitment strategies for environmental health studies: common partnerships and in-person engagement can meaningfully improve participation and representativeness. However, community-engaged recruitment’s higher radon test return rate was paired with lower survey completion; future community-engaged efforts may benefit from strategies that narrow this gap, such as completing surveys on-site or using real-time reminders for participants enrolled at events. Furthermore, these results should be interpreted in light of the study’s single-state setting, modest subgroup sample sizes, and differences in how recruitment success was measured across strategies; future research should examine measures that allow more direct comparison of recruitment effectiveness across approaches. This comparison highlights the critical role of community engagement in advancing equitable participation in studies examining environmental exposures and climate-related health risks. Continued investments in community-driven research infrastructures will be essential for strengthening trust, improving data representativeness, and informing policies that reduce radon exposure and lung cancer disparities in North Carolina.

## Figures and Tables

**Table 1:** Average demographic, radon exposure, and EPA zone characteristics of target counties for each region in 2024.

CLOVER Region	Avg. Black/African American %	Avg. Hispanic/Latino %	Avg. American Indian %	Avg. Radon Ranking (pCi/L)	EPA Zone Designations
Mountains	2.0	6.6	2.49	378.6 ^1^	1, 2
Piedmont	22.8	12.8	1.60	56.3	1–3
Coastal Plains	42.4	8.6	1.79	9.6	2, 3

^1^ The mean radon level reported for the mountain region is influenced by an outlier value recorded for Wilkes County (3200 pCi/L), based on NC Department of Health and Human Services county-level data available at the time of analysis (March 2024); this source has since been updated and no longer reports this value [17].

**Table 2:** Participant demographics from usual recruitment vs. community-engaged recruitment.

	ABS Recruitment n (%)	Community-Engaged Recruitment n (%)	Total n
Consented ^1^	86 (1.3)	150 (10.2)	236
NH Black	32	66	98
Hispanic/Latino	3	8	11
American Indian	1	12	13
Surveys Completed	63 (73.3)	82 (54.7)	145
NH Black	26	28	54
Hispanic/Latino	3	6	9
American Indian	0	12	12
Radon Test Results	19 (22.1)	67 (44.7)	86
NH Black	7	27	34
Hispanic/Latino	1	3	4
American Indian	0	4	4

^1^ NH Black, Hispanic/Latino, and American Indian participants combined represented 41.9% (36/86) of ABS-recruited and 57.3% (86/150) of community-engaged consented participants.

**Table 3:** Sociodemographic characteristics of surveyed participants.

n = 145	Mean SD	Minimum–Maximum
Age (y)	49.8 ± 15.8 n	18–93 %
Sex		
Female	94	64.8
Male	47	32.4
Other	4	2.8
Race and ethnicity		
African American, Black	54	37.2
American Indian or Alaska Native	12	8.3
Asian	34	23.4
Hispanic/Latino or Spanish Origin	9	6.2
White	36	24.8
Educational level		
Middle school or less	2	1.4
Some high school	4	2.8
High school	14	9.7
College, technical, or associate degree	36	24.8
University or above	86	59.3
Not reported	3	2.1
Employment status		
Employed (part-time or full-time)	93	64.1
Not employed/homemaker	11	7.6
Retired	26	17.9
Student	2	1.4
Temporarily unable to work	2	1.4
Other	11	7.6
Health insurance		
Private insurance (with or without state insurance)	83	57.2
Health Maintenance Organization (HMO) plan	5	3.4
State insurance (Medicare, Medicaid)	47	32.4
Uninsured	10	6.9

## Data Availability

The datasets presented in this article are not readily available because the data are part of an ongoing study and analyses are still in progress. Requests to access the datasets should be directed to the corresponding author.

## Supplementary Materials

The following supporting information can be downloaded at https://www.mdpi.com/article/10.3390/ijerph23081072/s1: Table S1: County selection criteria; Table S2: Community partners by organization type and county.
