# Supplementary material for: Examining Community Engagement Strategies Used in the Climate Impact on Lung Cancer via Exposure to Radon (CLOVER) Study
Source: Int J Environ Res Public Health. 2026 Aug 18;23(8):1072. doi: 10.3390/ijerph23081072 (PMC13513679; doi:10.3390/ijerph23081072)
Supplement: Supplementary file 1 [file ijerph-23-01072-s001.zip › ijerph-4437672-supplementary.pdf]

Supplementary Material

Table S1. County Selection Criteria.

Criterion Type	Threshold	Rationale
EPA Zone	Zone 1	Identifies high radon risk areas
Regional Radon Levels		
Mountains	>100 pCi/L	Captures geographic variation in exposure
Piedmont	> 75 pCi/L	
Coastal Plains	> 50 pCi/L	
Demographic Populations		
NH Black	>40%	Prioritize populations with lung cancer disparities
Hispanic/Latino	>15%	
American Indian	> 2.9%	

Table S2. Community partners by organization type and county.

Organization Type	Partner Name	County
Tribal	Lumbee Tribe of North Carolina	Robeson
	Coharie Tribe	Sampson
	Haliwa-Saponi Tribe	Halifax
	Guilford Native American Association	Guilford
	Occaneechi Band of the Saponi Nation	Alamance
Faith-Based	White Plains Methodist Church	Wake
	St. Paul's Christian Church	Wake
	Moore's Chapel African Methodist Episcopal Zion Church	Lenoir
	The River Church	Durham
Community-Based	African American Cultural Festival of Raleigh and Wake County	Wake
	El Centro Hispano	Wake
	The Consulate of Mexico (Raleigh, NC)	Wake
	County Outreach Ministries	Caswell
	Food Bank of the Albemarle	Pasquotank
	Vance County Senior Center	Vance
Governmental/Institutional	Hoke County Health Department	Hoke
	Brunswick County Environmental Health	Brunswick
	University of North Carolina at Pembroke	Robeson
